# A computational framework to support the treatment of bedsores during COVID-19 diffusion

**DOI:** 10.1007/s12652-022-03886-x

**Published:** 2022-05-27

**Authors:** Ferdinando Di Martino, Francesco Orciuoli

**Affiliations:** 1 Dip.to di Architettura, Università degli Studi di Napoli Federico II, Via Toledo 402, Napoli, Italy; 2 Dip.to Scienze Aziendali - Management & Innovation Systems, Università degli Studi di Salerno, Via Giovanni Paolo II, 132 Fisciano, SA Italy

**Keywords:** COVID-19, Pressure ulcers, Deep learning, Augmented reality, Pattern matching, Image segmentation, Edge detection

## Abstract

The treatment of pressure ulcers, also known as bedsores, is a complex process that requires to employ specialized field workforce assisting patients in their houses. In the period of COVID-19 or during any other non-trivial emergency, reaching the patients in their own house is impossible. Therefore, as well as in the other sectors, the adoption of digital technologies is invoked to solve, or at least mitigate, the problem. In particular, during the COVID-19, the social distances should be maintained in order to decrease the risk of contagion. The Project Health Management Systems proposes a complete framework, based on Deep Learning, Augmented Reality. Pattern Matching, Image Segmentation and Edge Detection approaches, to support the treatment of bedsores without increasing the risk of contagion, i.e., improving the remote aiding of specialized operators and physicians and involving inexperienced familiars in the process.

## Introduction

In this research we propose a framework that allows us to assist patients suffering from pressure ulcers (Hyun et al. 2019) during pandemic periods in which the patient’s home care can become critical and it is necessary that the patient, being a weakly affected subject, is not placed in contact with others, for fear that they may contract the virus disease. In this context, the number of home assistance for the patient must be limited to emergencies only and solutions are needed that guarantee patient assistance remotely. To analyze the state of his injury, the patient himself or a relative can photograph the lesion using a mobile phone (Cicceri et al. 2020). An AR-based Mobile App can be executed to classify and measure bedsores and receive diagnostic indications on the treatments to be performed. Furthermore, the photo of the pressure sore is sent and archived in the medical record on a server where an expert team can analyze it in detail and decide on any actions to be taken or further prescriptions to be sent to the patient. Our framework is configured as a workforce management system, thanks to which it is possible to obtain the right trade-off between the costs of assistance and the benefit for the patient. It allows a correct management of emergency situations such as the current COVID-19 pandemic crisis (Watson et al. 2020), in which it is necessary to satisfy the need to adopt social distancing, when possible, without reducing the priority of the patients’ health status. Furthermore, the functionalities of the framework are in line with the principles of Society 5.0 (Fukuyama 2018), which put man at the center and insist on technological innovation (Artificial Intelligence, Big Data, etc.) as a support aimed, among other things, for the inclusion, sustainability and improvement of the quality of life. The patient manages to be more and more actively involved in his care path by providing useful data to improve his quality of life and allowing public and private operators to provide increasingly sustainable services.

Recently some authors have proposed frameworks that implement AI models to support the analysis and treatment of patients during COVID 19 emergencies. In (Murugappan et al. 2021) a deep learning-based neural network in parallel architecture has been proposed for the classification of lungs healthy, pneumonia-affected lungs produced by COVID-19 disease and pneumonia-affected lungs produced by other diseases. In (Kaur et al. 2021) a new image processing-based technique for the health care systems is applied to detect COVID-19 infection from chest X-Ray images, in which a Support Vector Machine classifier is used to classify and detect COVID-19 infection into four different classes.

The proposed framework is composed of the AR-based Mobile App to support the patient for the classification of the pressure ulcer and the prescription of any treatments and the Back-end Analysis Dashboard used by the medical team for the detailed analysis of the pressure ulcer. In the next paragraph the overall framework is described.

## Overall framework

**Fig. 1:**
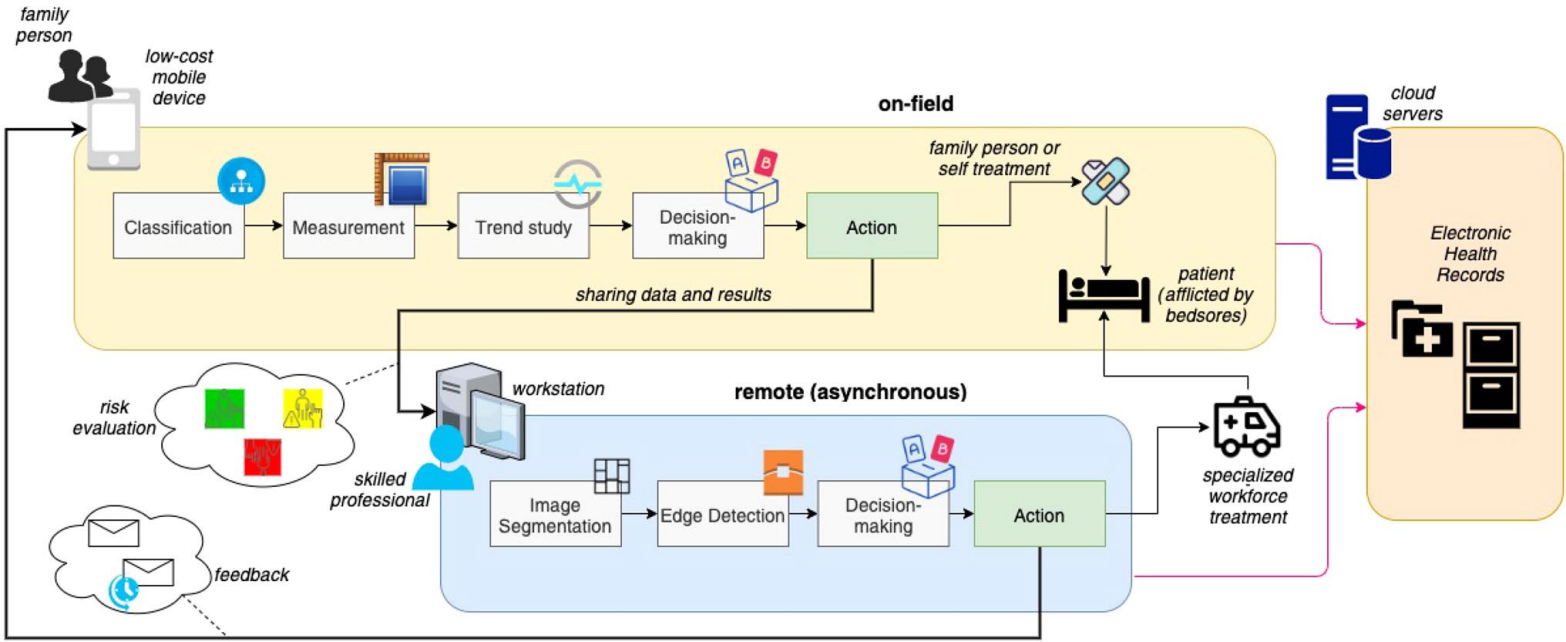
Sketch of the overall architecture

Extensive wound care is needed to prevent that the bedsore leaves the early stage to reach an advanced stage (Arora 2019). Typically, skilled professionals use consolidated approaches and obtain very promising results. The challenge here is to obtain satisfactory results when providing assistance to patients without deploying professional skilled field workforce. In particular, the proposed framework has been defined to provide solutions for scenarios in which it is needed to reduce the number of intervention where specialized operators go to the patients’ house. Lockdown windows, due to COVID-19 pandemic, provide a concrete example of the aforementioned scenarios. In such windows the number of domiciliary care interventions are drastically reduced or even completely reset. Another possible concrete scenario is represented by situations in which health facilities decide to decrease the costs. In both cases, it is strongly required to maintain stable the quality of service level. Therefore, new technology solutions must be proposed to achieve the aforementioned goals. In this context, as anticipated before, the proposed framework provides an integrated solution described in the overall picture of Fig. 1. From the architectural viewpoint, the proposed solution includes two main co-operating software components, also interacting with a cloud server providing *Electronic Health Records (EHR)* storage services. The first component is an AR-based Mobile App providing a set of functionalities allowing non-professionals caregivers to classify and measure bedsores and receive suggestions related to adequate and immediate care. The second component is a Desktop Application providing a set of functionalities allowing professional physicians to go deep in details with respect to the bedsore assessment and the planning of a care pathway. From the flow of actions viewpoint, the proposed solution tries to improve the cost-benefit ratio by balancing the use of the AR-based Mobile App and that of the Desktop Application with the idea in mind that the first one is used by patient’s familiars (the involvement of low-skilled people leads to cost reduction) and the second one is used by experienced physicians (the involvement of high-skilled people leads to cost increase). Hence, the approach is to adopt the AR-based Mobile App more frequently in order to identify simple cares which can be provided by the patients’ familiars without involving specialized workforce. The usages of the mobile app are continuously traced and periodically assessed by high-experienced physicians (through the Desktop Application) who can decide to intervene (e.g., by sending specialized workforce on-the-field) or to agree with the work of the mobile app. High-experienced physicians could be also directly invoked if the AR-based Mobile App detects a critical situation that must be evaluated by professionals. All the outcomes of the two main components are stored on the cloud storage in standard formats for feeding the EHR. In the next two Sections, the Ar-based Mobile App and the Desktop Application will be described in details.

## AR-based Mobile App

The idea underlying this component is twofold: (i) defining the model of a Clinical Decision Support System (CDSS) targeted to low-experienced or inexperienced people (family members or the patient herself) helping patients with bedsores, and (ii) designing and implementing an adequate architecture allowing the aforementioned users to use only low-cost devices (smartphones or tablets). Such low-cost devices have the advantage to provide user interfaces which are well known to the identified users. Thus, the aforementioned CDSS has been implemented as an Mobile App (AR-based^1^) and deployed on Android devices.

### Clinical decision support system

The CDSS helps the user to choose a *suitable course of actions* related to the bedsore treatment. In this context, the final decision should be made only when a set of high-level information has been correctly inferred from (raw) data. Such information is related to: (i) the stage in which the bedsore can be classified, (ii) additional attributes of the bedsore (skin conditions in the lesion area, extension of deep tissue damage, presence of necrosis), (iii) dimension of the bedsore and iv) temporal evolution of the bedsore. Typically (without using the proposed CDSS) the above information is derived by field operators through the exploitation of their skills and experience in analysing what they perceive from direct observation of the bedsore. Moreover, textual description included in reports of past examinations are used to build a temporal evolution that is also enriched by interviewing the patient. In particular, EPUAP (NPUAP for USA) (Edsberg et al. 2016; Taradaj 2017) classification is based on the identification of the stage in which the bedsore currently is. Being aware of the correct bedsore stage is fundamental to understand how to act directly or asking for the surgical consultation.

**Fig. 2:**
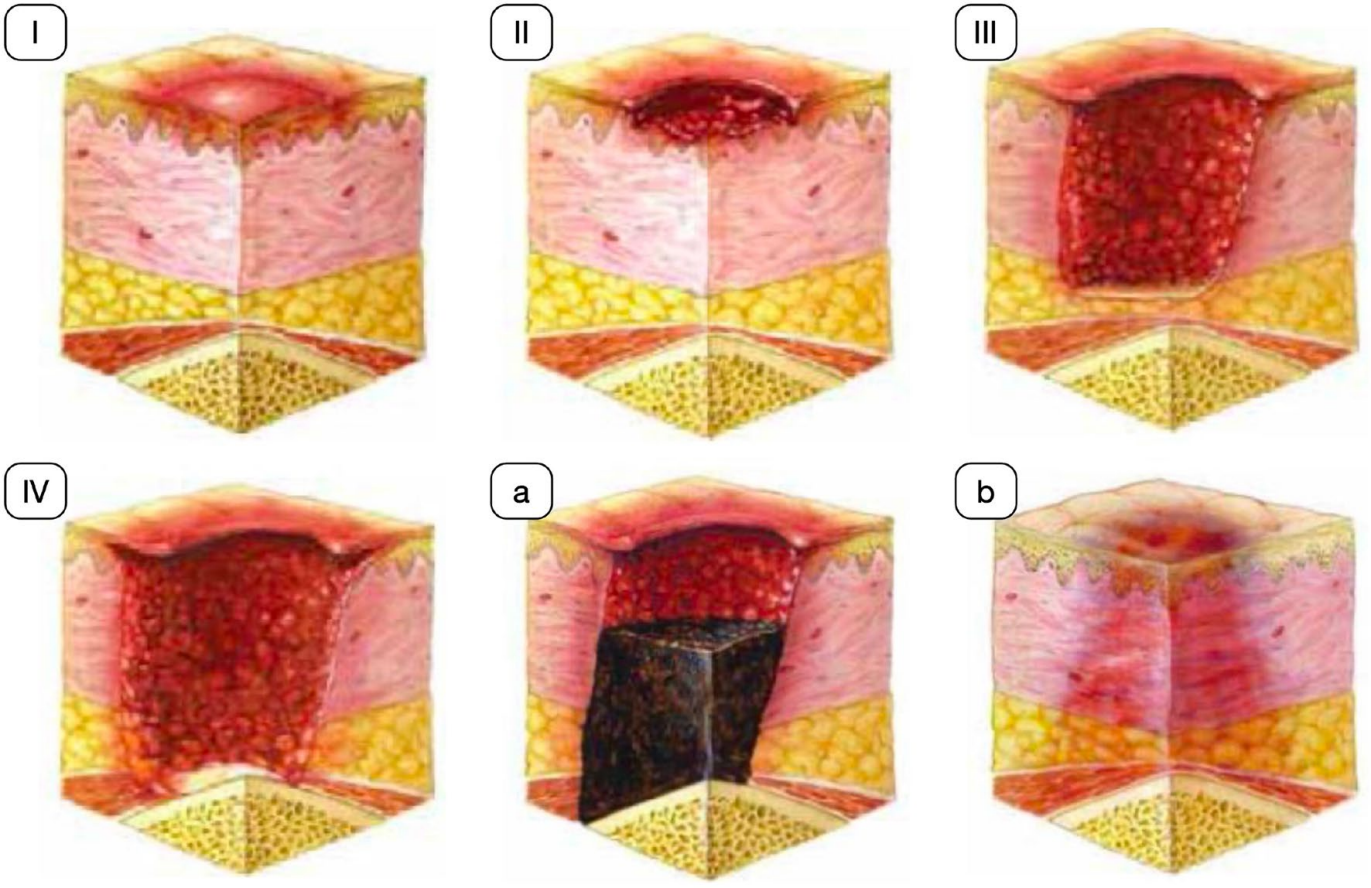
Stages for EPUAP/NPUAP. (Source: Italian version of (Kottner et al. 2019))

More in detail, the EPUAP scale consists of four stages: (I) non-blanchable erythema, (II) partial thickness skin loss, (III) full thickness skin loss, and IV) full thickness tissue loss. Two additional stages have been added (unstageable: depth unknown and suspected deep tissue injury: depth unknown) (Kottner et al. 2019). Figure 2 graphically reports such stages.

The CDSS is functionally decomposed in three main components: *bedsore classifiers*, *bedsore measurement tool* and *bedsore time machine*. More in detail, the first one is a set of four classifiers able to determine the NPUAP/EPUAP stage and the further three attributes of the bedsore whose image is captured by the smartphone/tablet camera. The automatic classification results (produced by applying a Deep Learning approach) (Vijayalakshmi and Jose 2018; Abubakar et al. 2019) are graphically registered and attached on the bedsore image by means of an Augmented Reality engine (invoked by the App). Through this component, the user can receive a first support for the assessment task in which she is involved in without switching on other tools or systems, in other words, the AR-based App acts in a situated way. The second component is a tool that supports the user in making one or more measures of the bedsore dimensions. Such component does not force the operator to use additional devices or changing the main screen of the application. Also in this case, Augmented Reality is the key enabler. The third component allows the operator to travel along the timeline to get past examination results in terms of measurements and classification results to sustain her comprehension regarding the evolution of the bedsore.

### Architectural and implementation issues

The Android AR-based App has a simple architecture: Unity3D^2^ (with C# as scripting language) and EasyAR^3^ are the technologies adopted for the implementation of the App front-end, the local storage and the *Time Machine* inner component (namely Bedsore Time Machine). PyTorch^4^ is the Deep Learning technology used to implement the *Classifiers* deployed as a remote component, i.e., to train the classification models (namely Bedsores Classifiers), and Open CV^5^ is the Computer Vision technology exploited to realize the *Measurement Tool* also deployed as a remote component (namely Bedsore Measurement Tool). Remote components live within Web Services realized through Python Flask^6^ and exchange data with the main App by using the JSON^7^ portable format. There is also the possibility to plug further services in order to connect the App with external systems like, for instance, electronic health records (EHR), patient relationship management (PRM), and so on. The Web Service invocations, within the App, are realized through HTTP requests implemented by using C# scripts.

#### Classifiers

**Fig. 3:**
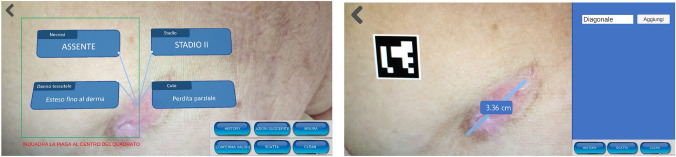
Two screenshots of the prototype. (Source: (Orciuoli et al. 2020))

Four classifiers are built by applying a Deep Learning approach over a training set of bedsore photos^8^. Such classifiers, once embedded into the AR-based App (see Fig. 3), provide the inexperienced user with suggestions related to the identification of the ulcer with respect to a set of parameters. The dataset has been divided into the training set (75%) and the test set (25%) only after having pre-processed the whole image set (opportunely augmented) to obtain PNG images of 192 × 192 normalized pixels. With respect to the Deep Neural Network architecture, in order to deal with a poor training set, the idea was to start from a pre-trained network. Therefore,

MobileNet V2^9^ has been adopted to define the backbone of the deep neural network architecture. MobileNet V2 is a convolutional neural network that can be customised and fine tuned for enabling the training task without a huge amount of samples, with low resolution images, using low-cost hardware and providing results with high levels of accuracy. MobileNet V2 is much faster than the regular convolution with approximately same result. Each layer of MobileNet V2 has batch normalization and ReLU as activation function. However, the output of the projection layer does not have an activation function (Michele et al. 2019). The proposed approach employs four MobileNet V2 networks (one for each bedsore attribute to consider for the classification task) and replaces, for each network, only the last layer (a classifier with 1000 classes supported) with a classifier supporting only the number of classes needed to handle the specific bedsore attribute. For instance, in the network for the classification related to the attribute *NPUAP/EUPAP stage* the classifier supports four classes (stage I, stage II, stage III, stage IV). Moreover, the approach foresees a further step represented by the application of transfer learning (a machine learning method used to adjust an original model trained to accomplish an original task in order to execute a second different task). In practice, for the Bedsore Classifiers, only the last layer of each network has been trained by using the bedsore training set. The remaining layers of all networks have been pre-trained by using ImageNet^10^, i.e., an image database organized according to the WordNet hierarchy (currently only the nouns), in which each node of the hierarchy is depicted by hundreds and thousands of images. Lastly, the fine tuning step has been accomplished by means of the trial-and-error practice.

#### Measurement tool

The Bedsore Measurement Tool (see Fig. 3) allows the user to measure the dimensions of the ulcer by using the same device and the same App exploited for the lesion classification and the other functionalities during the same working situation. In general, a bedsore has not a regular shape so there could be the need to allow and store several measures (diagonal, height, width, etc.). The field operator can use a simple graphical tool to draw lines, in Augmented Reality mode, directly on the bedsore image (captured by the device camera) and measure the length of each line. The issue to face is to convert the above measures from pixels to centimeters (or other conventional units) but this operation depends on the position and rotation of the camera with respect to the bedsore. The proposed approach consists in using a small 2D marker to obtain a scale factor for converting pixels in centimeters. In particular, the adopted marker, namely *ArUco Marker* (Garrido-Jurado et al. 2014), must be printed and positioned near the bedsore in a way that it is possible, for the device camera, to capture a scene in which the bedsore is shown together with the marker and provide it to the device display. The field operator can draw more lines over the bedsore on the display (step 1). Each line represents a dimension to measure.

## Back-end analysis dashboard

The back-end analysis Dashboard has been implemented to optimize the recognition processes of the shape of the lesion in the photo taken of the patient. It allows the expert, be he a doctor or nurse following the patient, to catalog and analyze in detail the image of the acquired lesion. The image of the pressure ulcer, which can also be taken by a cohabitant of the patient or by the patient himself using his mobile phone, and transmitted on the server and is stored in the patient’s medical record by the expert doctor who, after analyzing it, can decide if further analysis is needed and image processing in order to detect the injured part and examine it.

The back-end analysis services concern the image segmentation and edge detection functionalities that allow to extract from the photo and analyze the shape of the wound. The idea behind the dashboard experimentation is to provide support for patient injury analysis and monitoring of its evolution by providing a user friendly interface that allows the use of advanced back end functions of image segmentation and edge detection, allowing you to archive the structure and shape of the outline of the wound in the patient’s medical record and allow it to follow its evolution over time. The main advantages of the dashboard are:

- the possibility of monitoring the state of the wound even in pandemic emergency situations where it is not possible for the nurse or doctor to access the patient’s home for specific checks. In fact, the patient himself or his relatives can take the photo of the lesion and transmit it to the medical team who, in case of need, can further examine it by performing the image segmentation and edge detection functionalities of the back-end analysis dashboard and then quickly decide the actions to be taken;
- the high ductility of the framework. For example, the expert can decide to perform image segmentation functions, which are computationally expensive, on a compressed image of the patient’s photo, if he decides that the copied image contains all the information necessary to examine the shape of the wound, or consider vice versa, that even a slight lossy compression of the image involves the elimination of significant information and the decision to segment the original image. In the same way, the expert can decide to extract the edges of the wound directly from the original image, or from a segmented image, if in the latter the shape of the wound is more evident.

**Fig. 4:**
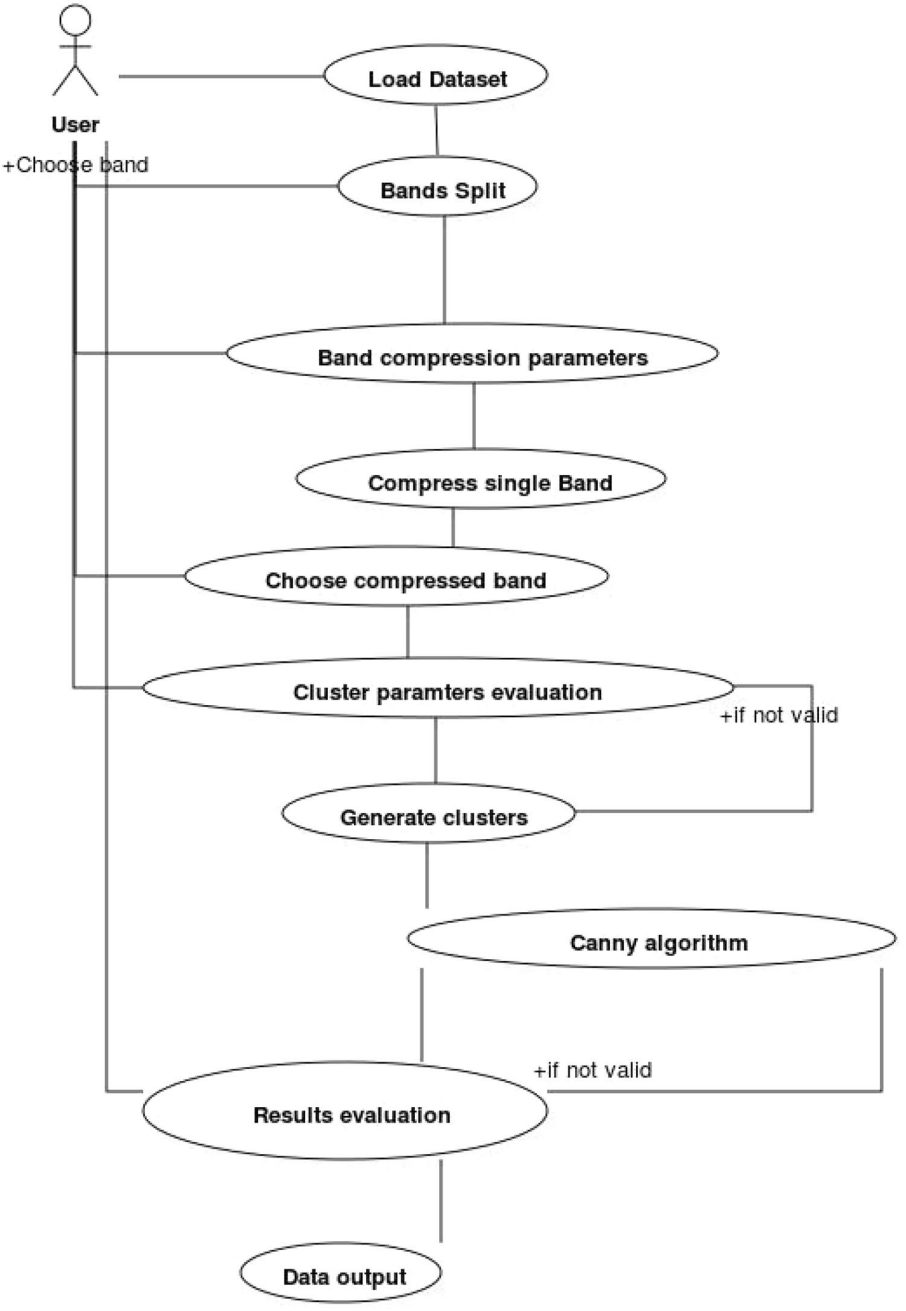
Use case diagram of the Back-end functionalities

Before being processed, the image is broken down into the three bands Red (R), Green (G) and Blue (B) and the image in each band is analyzed individually as the shape and size of the lesion can be different and differently recognizable in the 3 bands. After analyzing the image in a specific band, the expert can eventually decide to compress it using an image compression lossy algorithm, in order to subsequently apply the image segmentation algorithm directly to the compressed image, considerably reducing the execution times. The block-partitioning bi-dimensional Fuzzy Transform method (Di Martino et al. 2008; Di Martino and Sessa 2007) is applied to compress the image. The expert can decide to use the grey-level source image in a band or a correspondent compressed image in the segmentation process in which the input image is segmented in C clusters using the FGFCM algorithm (Di Martino et al. 2010). The result of the segmentation consists of C images in which the gray level value is obtained by denormalizing the degree of belonging to the cluster. Each of the C images can be further analyzed for the purposes of extracting the contours of the injured zone by applying the Canny edge detection algorithm (Canny 1986). Each of the three main functions of compression, segmentation and edge detection includes a set of functions to support the expert for choosing the optimal parameters to use and checking and appropriately archiving the results obtained. The Use Case diagram in Fig. 4 shows the use cases of the functionalities of the system; in it all the functionalities included in the dashboard are schematized.

**Fig. 5:**
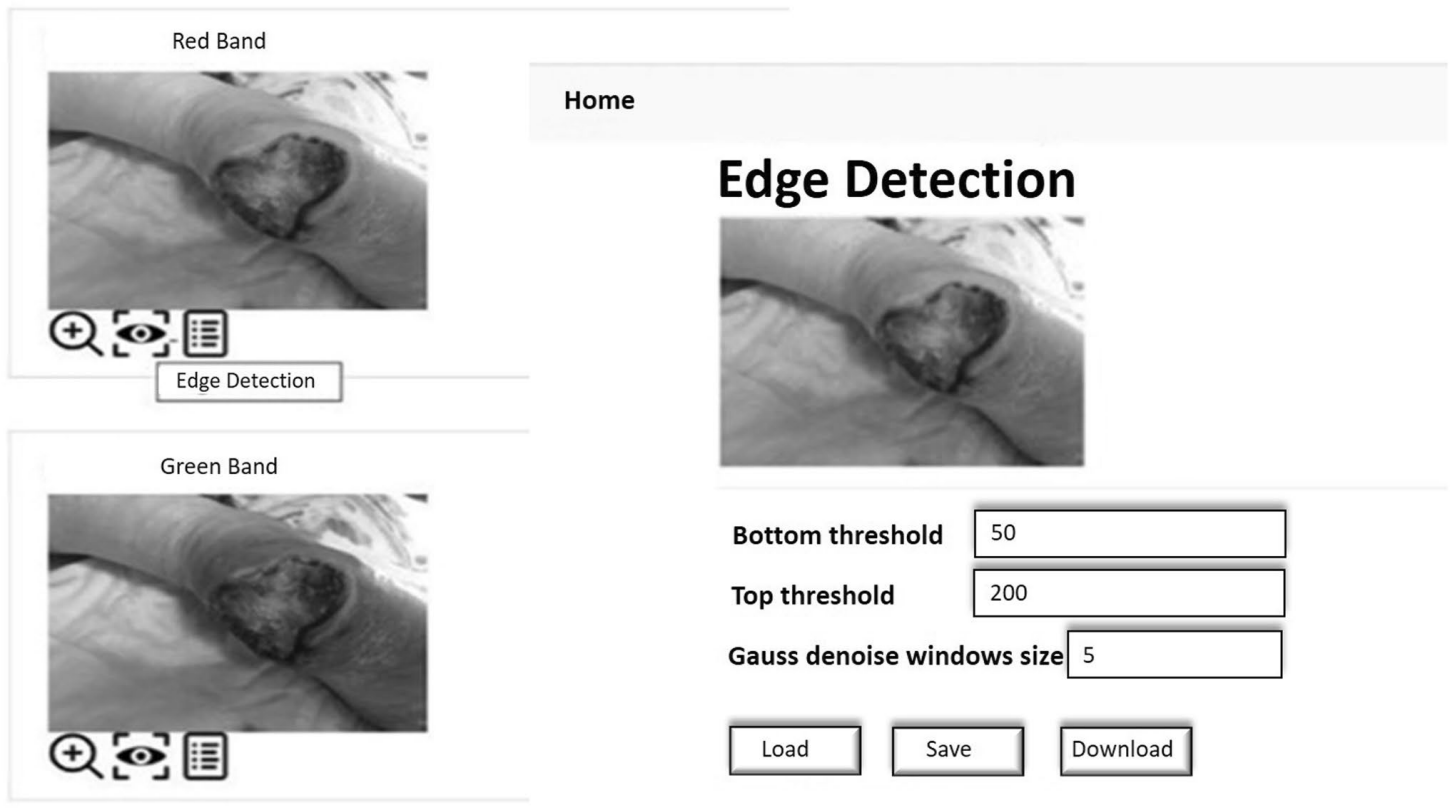
Example of execution of the edge detection functionality. Edge detection is performed for the source image in the Red band

In Fig. 5 is shown an example of application of the edge detection functionality. The user decides to detect the injured area directly from the original image in the R band.

**Fig. 6:**
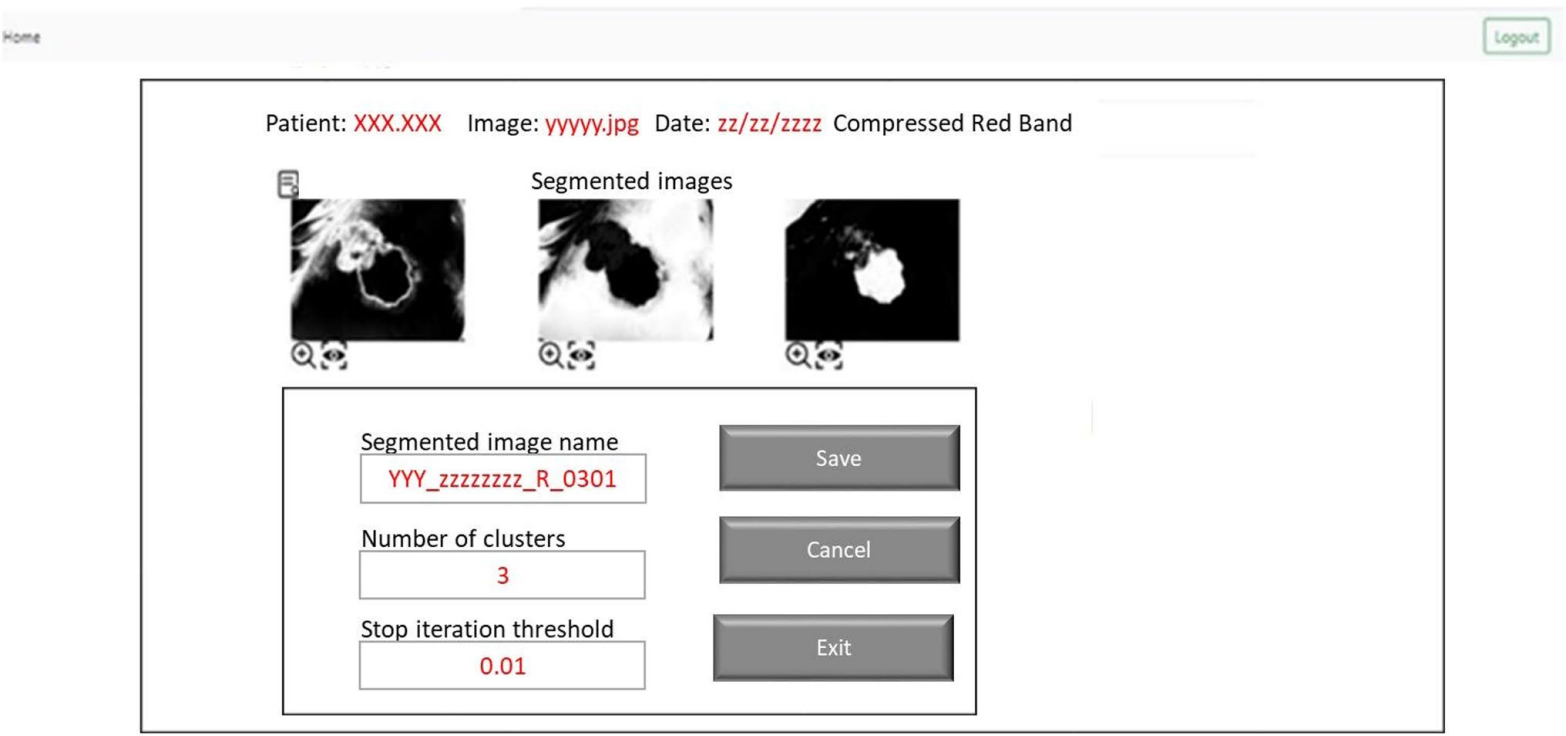
Example of results obtained executing the image segmentation functionality (the compressed image in the Red band is segmented in three clusters)

In Fig. 6 is show the result of the segmentation of a compressed image in the Red band. The image is segmented in three clusters and the three segmented resultant images can be analyzed in detail; the user can decide to execute the edge detection functionality of a segmented image (Di Martino et al. 2010).

## Results of the case study

The experimentation of the framework was carried out by analyzing the performance of the AR-based app and the back-end analysis dashboard separately. It was aimed at measuring the performance capabilities related to the AR-based lesson classification functionality and the back-end capabilities of image segmentation and lesion contour detection.

### Tests of the AR-based App

An experimentation activity has been carried out by means of the prototype of the AR-based App (see Fig. 3). In particular, the idea is to prepare and execute a laboratory test in which three inexperienced users exploit the AR-based App (installed on a 6-inches display smartphone) to be supported in making a decision related to a bedsore treatment. In particular, the experimentation is focused on three bedridden patients with pressure ulcers. The history of the three patients is pre-loaded into the App in order to enable also the *Bedsore Time Machine* functionality. Each history is constructed along three time points. For each point, bedsore photo, measurements and classification data have been provided through the aforementioned functionality. Furthermore, a new photo of the last evolution step of the ulcer for all patients has been provided. The three operators were asked to use the App and the photo and provide their assessment results and the selected course of actions for the bedsore treatment. Lastly, operators’ decisions and results have been compared to those of a third high-experienced operator to rate their quality. A total of 12 classification results (4 attributes for 3 patients), for each user, has been collected. The percentage of correct results (validated by the high-experienced operator) was around 78% (81% for the first user, 75% for the second user and 77% for the third user).

### Tests of the back-end analysis dashboard

**Fig. 7:**
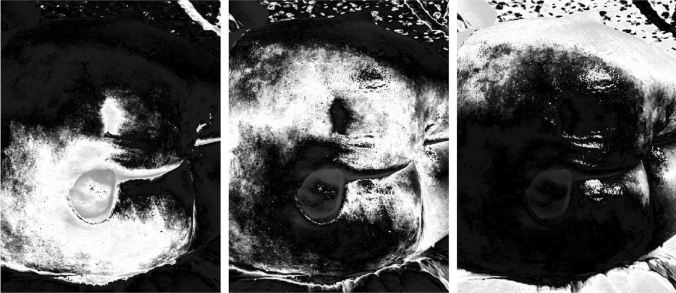
Segmentation results of a source image in the R band

To compare the quality of the results of the image segmentation feature, the image segmentation process was first performed for the input image of the lesion in each of the 3 bands and subsequently performed on each of the 3 bands of the compressed image. with a specific compression ratio. The segmented images were first decompressed in order to obtain images of the same size as the original image and then were compared with the corresponding segmented images. The indicator used to measure the quality of the segmented image obtained from the compressed image using the Fuzzy Transform technique is the Peak Signal to Noise Ratio (PSNR), a well-known measure of image quality compared with the original image. In this test it is used to check how much the quality of an image produced by the segmentation of the compressed image has decreased in terms of performance compared to the corresponding segmented image obtained from the original image. In Fig. 7 are shown the results of the segmentation process applied on a source image in the R-band.

**Fig. 8:**
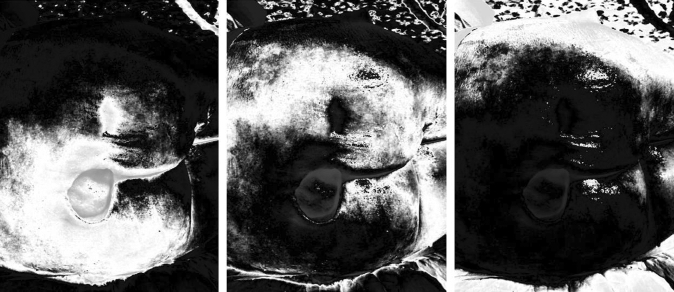
Segmentation results of a source image in the R band compressed with a compression rate 0.25

Figure 8 shows the results of the segmentation process applied on this image in the R band compressed using a compression rate 0.25.

**Fig. 9:**
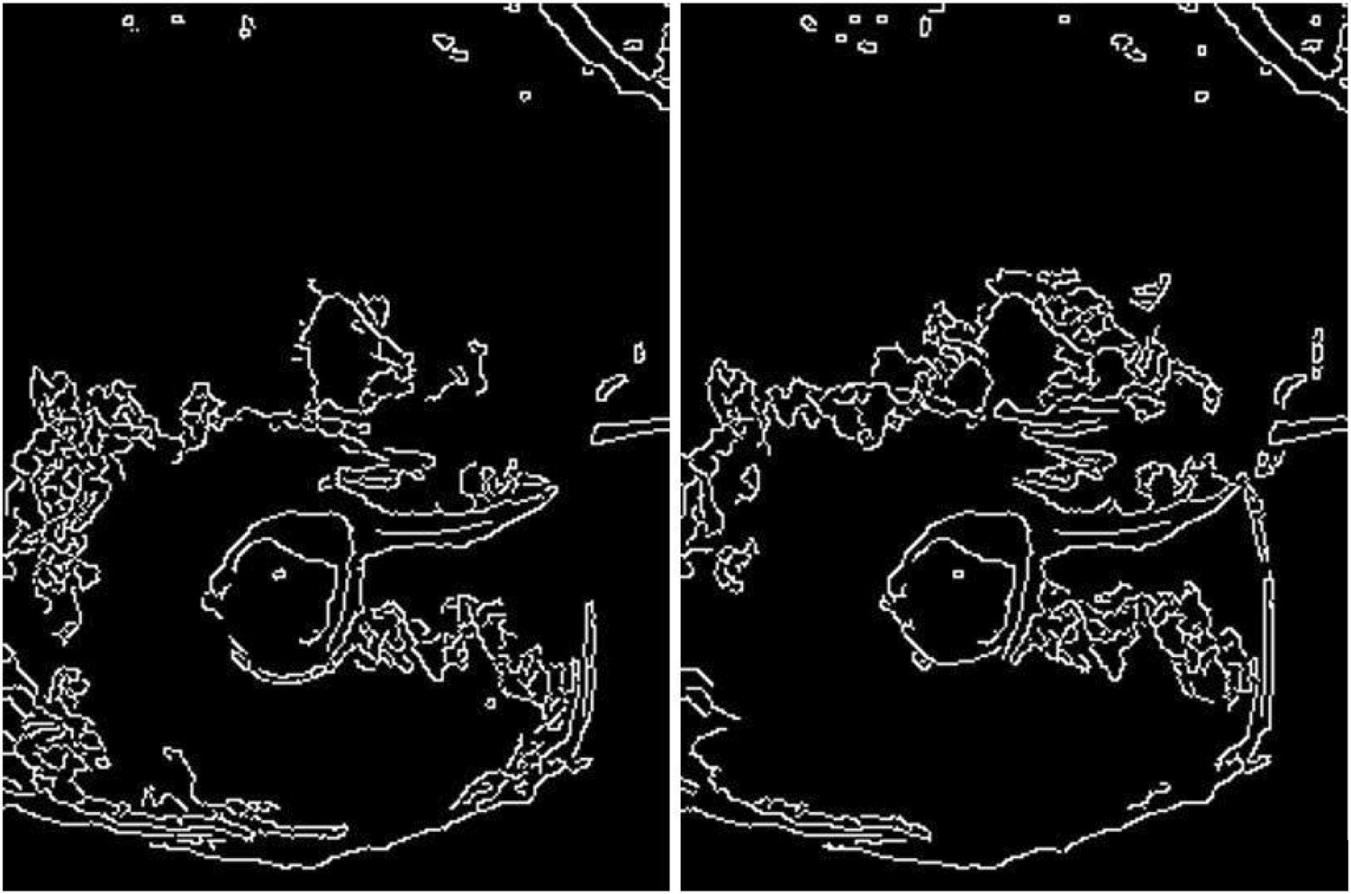
Comparison of edge detection results obtained from a segmented original image in the Red band and from the segmented corresponding image compressed using a compression rate 0.25

All the trends of the PSNR index as the compression rate varies show an almost exponential decrease of the PSNR for strong compression (compression rate values lower than 0.1). This result is supported by the fact that for very strong compression the loss of information in the image is high and this leads to an ever increasing degradation of the quality of the segmented image. On the contrary, for not excessive compression (compression rate greater than 0.1) the PSNR in the three bands shows a linear trend with a low slope, indicating that, in this case, the segmentation results obtained from the compressed image can be considered of acceptable quality. To measure the quality of the results of the recognition of the contours of the pressure ulcer, the binary image resulting from the edge detection process obtained from the segmentation of a compressed image in a band is compared with that obtained from the corresponding segmented image obtained from the original image in that band. The two edge detection processes are performed by assigning in both cases the same values of the denoising window and of the minimum and maximum value of the hysteresis thresholds. The binary image derived from the segmentation of the compressed image is enlarged to the original image size before making the comparison. In Fig. 9 are shown the edge detection results of a segmented image obtained from the original image compressed in the R-band and the corresponding one obtained from the compressed image with a compression rate 0.25. The two binary images resulting from the edge detection process are very similar; this highlights that the compression of the original image with a compression rate 0.25 did not generate considerable losses of information in the segmented images.

To evaluate the quality of the resulting binary image obtained starting from the compression of the original image, the indicators accuracy, precision, recall (or sensitivity) and F1 score were measured by comparing the binary image obtained enlarged to the size of the original image with that obtained without performing compression. The trends of all four indicators as the compression rate varies show that the quality of the results remains acceptable for compression rates not lower than about 0.1. In fact, in these cases all four indicators have average values of no less than 70%. For greater compression (compression rate less than 0.1) the values of the 4 indicators decrease rapidly. The results of these tests confirm that for compression that are not excessively high, the quality of the images obtained downstream of the segmentation and edge detection processes after compressing the original images of the lesions using the fuzzy transform algorithm is acceptable. The trends in all three bands show a linear trend with a low slope for compression rates not lower, approximately, to the value 0.1.

## Conclusions

We propose a complete framework based on Deep Learning, Augmented Reality. Pattern Matching, Image Segmentation and Edge Detection approaches, developed during the research project Health Management Systems, aimed at the remote assistance of patients with pressure ulcers, during a non-trivial emergency periods in which reaching the patients in their own house is impossible. Our framework consists of an AR-based App that provides an inexperienced user such as the same patient or family members with an easy-to-use Clinical Decision Support System on their mobile phone to assist them in the treatment of bedsores and of a back-end analysis dashboard that allows remote detailed analysis of the image of the pressure sore sent by the patient and monitoring of its state over time. The results of experimental tests show that the framework provides good performance of the framework in terms of accuracy of results and execution times. We intend in the future to further improve the performance of the framework in terms of the accuracy of the classification and measurement of the bedsore and localization of the shape and structure of the bedsore in the image, and to increase the functionality of the framework with additional services that include complete management of the patient’s medical record, in-depth analysis of the evolution of the lesion.

## Footnotes

1 Providing Augmented Reality features.

2 https://unity.com/.

3 https://www.easyar.com/.

4 https://pytorch.org/.

5 https://opencv.org/.

6 https://palletsprojects.com/p/flask/.

7 https://www.json.org/json-it.html.

8 Provided by INNOVA MAGALDI srl, a project partner.

9 https://machinethink.net/blog/mobilenet-v2/.

10 http://www.image-net.org/.
